# Morphological and Immunohistochemical Characterization of Canine Osteosarcoma Spheroid Cell Cultures

**DOI:** 10.1111/ahe.12190

**Published:** 2015-08-19

**Authors:** C. Gebhard, C. Gabriel, I. Walter

**Affiliations:** ^1^ Institute of Anatomy, Histology and EmbryologyUniversity of Veterinary MedicineViennaAustria; ^2^ Vienna VetCore Facility for ResearchUniversity of Veterinary MedicineViennaAustria

## Abstract

Spheroid cell culture emerges as powerful *in vitro* tool for experimental tumour research. In this study, we established a scaffold‐free three‐dimensional spheroid system built from canine osteosarcoma (OS) cells (D17). Spheroids (7, 14 and 19 days of cultivation) and monolayer cultures (2 and 7 days of cultivation) were evaluated and compared on light and electron microscopy. Monolayer and spheroid cultures were tested for vimentin, cytokeratin, alkaline phosphatase, osteocalcin and collagen I by means of immunohistochemistry. The spheroid cell culture exhibited a distinct network of collagen I in particular after 19‐day cultivation, whereas in monolayer cultures, collagen I was arranged as a lamellar basal structure. Necrotic centres of large spheroids, as observed in 14‐ and 19‐day cultures, were characterized by significant amounts of osteocalcin. Proliferative activity as determined by Ki‐67 immunoreactivity showed an even distribution in two‐dimensional cultures. In spheroids, proliferation was predominating in the peripheral areas. Metastasis‐associated markers ezrin and S100A4 were shown to be continuously expressed in monolayer and spheroid cultures. We conclude that the scaffold‐free spheroid system from canine OS cells has the ability to mimic the architecture of the *in vivo* tumour, in particular cell–cell and cell–matrix interactions.

## Introduction

Osteosarcoma (OS) is the most common primary bone tumour in adolescents and young adults (He et al., 2014; Xing et al., 2014). OS frequently arises in the skeletal region at the medullary side of long bones as well as in lower numbers at the extraskeletal region (Klein and Siegal, 2006; Broadhead et al., 2011; Gill et al., 2013; Luetke et al., 2014). This aggressive bone tumour is combined with severe clinical symptoms such as joint pain, heavily decreased mobility, fracture and metastatic spread (Picci, 2007; Clark et al., 2008). Considerable similar tumour characteristics were reported between human and canine OS regarding tumour behaviour, clinical symptoms, histology and molecular alterations (Withrow and Khanna, 2010; Rowell et al., 2011; Osborne and Khanna, 2012; Rankin et al., 2012). Human and canine OS share a high tendency to form metastases, 80% of them developing in the lung (Posthumadeboer et al., 2011). During the last decades, no significant improvement in human and canine OS therapies has occurred. Hence, there is a real demand for new approaches such as molecular targeted therapies (Broadhead et al., 2011; Haddox et al., 2014). Promising biomaterials are tumour cell‐based constructs particularly of three‐dimensional (3D) architecture studying the approximate *in vivo* tumour physiology (Pampaloni et al., 2007). In contrast, monolayer cultures are maintained as flattened cells adhered on plastic surfaces. These two‐dimensional (2D) models mirror an unnatural morphology and are therefore likely to have considerable influence on drug response. Nowadays, there is an increasing awareness of these drawbacks of 2D cell cultures (Hyman and Simons, 2011; Tan et al., 2011; Prideaux et al., 2014).

Numerous attempts have been made to develop 3D cell culture models for bridging the gap between cell‐based assays and animal studies to reduce experimental uncertainties arising from monolayer cultures and hence the cost of subsequent drug screening processes. It has been shown before that 3D grown cells are more valid targets compared to 2D monolayers for drug testing. Tan et al. (2013) reported the OS drug resistance of SaOS2 and U2OS cells grown as 3D scaffolds. Differences in pharmacological kinetics are likely to be due to a diffusion process which is obviously better reflected in *in vivo*‐like 3D models than 2D monolayers. Therefore, the 3D tumour model has the ability to reliably mimic a more appropriate *in vivo* situation including the naturally 3D architecture, cellular junctions and characteristically longer diffusion distances.

It is obvious that the results obtained from experimental *in vitro* approaches in tumour research are highly dependent on the quality of the used cell culture method. Therefore, the development of applicable cell culture models is desirable. Much effort has been made to reach suitable 3D cell culture models including organ explants or various scaffolds that support specific cell survival, growth and differentiation conditions (Page et al., 2013). Organ explants are tissue‐consuming, short‐lived and therefore inappropriate for several experimental approaches such as target validation or drug screening tests. Spheroids consist of aggregated cells with similar characteristics to *in vivo* tumour tissue (for review, see Kunz‐Schughart et al., 2004; Fennema et al., 2013). They represent a complex 3D network of cells with tight cell–cell and cell–matrix interactions. Spheroids are appropriate units for tissue reconstruction. Often, they are developed using 3D scaffolds consisting of porous materials or from extracellular matrix (ECM) proteins (Tan et al., 2011; Bartel et al., 2013; Lawrenson et al., 2013; Matsusaki et al., 2014). However, the application of scaffold materials alters cell morphology and physiology. Scaffold properties such as pore size, geometry and interconnectivity affect cell shape, cell–cell contacts, nutrient uptake and gene expression (Folkman and Moscona, 1978; Bissell, 1981; Bissell et al., 1999; Khademhosseini et al., 2006; Bae et al., 2014; Tome et al., 2014). A study of Nelson and Bissell (2006) supported the fact that tissue architecture properties directly influence cell physiology pathways. Moreover, the usage of artificial ECM components in 3D cell culture experiments has to be considered carefully. A widely used artificial ECM in spheroid cell culture experiments is Matrigel^™^. Vukicevic et al. (1992) demonstrated that different growth factors in the reconstituted Matrigel^™^ basement membrane were active in influencing the cellular network formation. In different studies, the authors indicate to draw attention in the interpretation of results concerning cellular behaviour and activity obtained in cell culture systems using artificial ECMs because of the huge number of unidentified components (Kleinman et al., 1986; McGuire and Seeds, 1989; Vukicevic et al., 1992). The potential role of these interactions on cell migration and matrix remodelling processes in 3D cell culture systems needs to be regarded (McGuire and Seeds, 1989). To overcome the problems with undefined artificial ECM, the aim of the study was to establish and characterize a 3D scaffold‐free spheroid cell culture model from the well‐defined canine OS cell line D17 (Legare et al., 2011) without substituted ECM components. The developed 3D culture system represents a flexible, straightforward and easy to reproduce OS cell culture model. Morphological characteristics of monolayers, as still mostly used in experimental approaches, have been compared with spheroid cultures to obtain a comprehensive view of the D17 OS cell biology. In respect of applying this 3D canine OS system in further experimental approaches, it is indispensable to collect as much data as possible to choose the most appropriate field of application. Based on this aim, we strongly seek to profoundly characterize the generated OS spheroid model. For analysis, we focused on different parameters such as proliferative activity, differentiation, matrix production, cytoskeletal markers, and ultrastructure morphology after defined culture time spans (2, 7, 14 and 19 day). Therefore, we applied light microscopy, histochemistry, immunohistochemistry and transmission electron microscopy (TEM) techniques to evaluate monolayer cultures and scaffold‐free canine OS spheroids.

## Materials and Methods

Canine OS cells (D17) were used for the 2D and 3D cell cultures and purchased from ATCC (CCL‐183, Wesel, Germany). As mentioned by the manufacturer, these cells were derived from an OS lung metastasis of an 11‐year‐old female dog.

### Monolayer cell culture

After thawing of D17 cells at 37°C for 2 min in a water bath, viability and cell number were determined using an automated cell counting system Countess^®^ (Thermo Fischer Scientific, Kalamazoo, MI, USA). D17 cells were seeded at a final concentration of 6 × 10³ cells/ml on 25‐cm² tissue flasks (Sarstedt, Nümbrecht, Germany) with sterile filtered standard medium. Components of the standard medium comprised 88% DMEM–high glucose (Lonza, Basel, Switzerland), 1% antibiotic–antimycotic solution (PAA, Pasching, Austria), 1% amphotericin B (Sigma‐Aldrich, Steinheim, Germany), 1% l‐glutamine (Sigma‐Aldrich) and 10% fetal bovine serum solution (Biochrom, Berlin, Germany).

Cells were transferred in 75‐cm² tissue flasks at a concentration of 2 × 10^6^ cells/ml and incubated in an atmosphere of 37°C and 5% CO_2_. Evaluation of cell growth was observed at intervals of 24 h using a tissue culture inverted microscope (Motic AE‐21; Motic, Wetzlar, Germany). Cells for the 2D cell culture model were harvested at two different time points: first after 2 day with up to 70% confluence and second after 7 day (>90% confluence). For harvesting the 2D samples, the cell lawn was detached mechanically from the bottom of the flask with a cell scraper (Greiner bio‐one, Kremsmünster, Austria).

## 3D Spheroid cell culture

The 3D architecture of spheroids was constructed without the addition of basement membrane components and ECM‐like biomaterials. The experimental design was conducted in non‐adherent round‐bottom 96‐well plates (Greiner bio‐one, Frickenhausen, Germany) for three different cultivation periods: 7, 14 and 19 day. Each approach was confirmed by four independent biological replicates (Rp 1–4). For spheroid growth, 10 ml D17 cell suspension of 4.0 × 10^5^ cells/ml standard medium was obtained from the monolayer cell culture. For the three different culturing periods, different dilutions of the cell suspension were used to avoid overgrowing of the D17 cells in the 96‐well formats. For spheroid growth, 7 day 150 *μ*l, for 14 day 100 *μ*l and for 19 day 50 *μ*l cell suspension were added per well and filled up with standard medium to a total volume of 250 *μ*l/well. For each experiment, 32 wells were used for the respective time point (7, 14 and 19 day). Medium was changed every second day until day 7, every day until day 14 and twice a day until day 19 due to the high metabolic activity of the growing spheroids. Spheroids were harvested using a plastic pipette of 2‐mm tip diameter, pooled in respective aliquots and centrifuged to cell pellets (2 min, 2.000 rpm) at room temperature (RT) to conduct further analyses.

The harvested cell culture material from both systems, 2D and 3D, was washed for two times with phosphate‐buffered saline (PBS; without Ca^2+^ and Mg^2+^; PAA) and subsequently once in 0.9% NaCl solution (Mayrhofer Pharmazeutika, Linz, Austria).

### Histochemistry

Cell pellets from 2D and 3D cell culture were fixed in 4% formaldehyde for 24 h at 4°C. After centrifugation (2 min, 2.000 rpm, RT), the supernatant was removed, and the cell pellet was embedded in Histogel^™^ (Thermo Fisher Scientific, Vienna, Austria) and subsequently embedded in paraffin (Histo‐Comp Medium; Vogel, Gießen, Germany) using an automated embedding system (Shandon‐Excelsior; Histocom, Wiener Neudorf, Austria). For histological analysis, the cell culture samples were cut in 3‐*μ*m serial sections, deparaffinized in xylene and rehydrated in a graded alcohol series. Histological staining was performed with haematoxylin and eosin (H&E) for general evaluation of the cell cultures, aniline blue to stain collagen fibres, safranin O to demonstrate cartilaginous ECM and periodic acid–Schiff (PAS) reaction for carbohydrates. All histochemical stainings were performed according to Romeis' protocols (Romeis et al., 1989).

### Immunohistochemistry

Serial sections of the formalin‐fixed and paraffin‐embedded samples (3 *μ*m) were mounted on aminopropyltriethoxysilane‐/glutaraldehyde‐coated slides. Endogenous peroxidase activity was blocked in case of horseradish peroxidase‐based immunohistochemistry with 0.6% H_2_O_2_ in methanol for 15‐min incubation at RT. A protein block was conducted to minimize unspecific binding of the primary antibody by 1.5% goat serum (DAKO, Glostrup, Denmark) in PBS for 30 min. The binding of primary antibodies (anti‐alkaline phosphatase, anti‐ezrin, anti‐metastasin S100A4, anti‐Ki‐67; details given in Table 1) was detected with the BrightVision (ImmunoLogic, Duiven, the Netherlands) secondary antibody system.

**Table 1:** Primary antibodies, sources, dilutions and pre‐treatments

Primary Antibody	Clone	Dilution	Pre‐treatment	Source
Alkaline phosphatase	Poly rabbit	1:100	Boil in 0.1 m citrate buffer 20 min (steamer)	Gene Tex, Irvine, CA, USA
Ezrin	Mono mouse, Clone 18	1:300	No pre‐treatment	BD Transduction Laboratories, San Jose, CA, USA
Metastasin (S100A4)	Poly rabbit	1:150	Boil in citrate buffer 20 min (steamer)	Thermo Scientific, Fremont, CA, USA
Ki‐67	Mono mouse, Clone 7B11	1:100	Boil in citrate buffer 20 min (steamer)	Invitrogen, Camarillo, CA, USA
Collagen I	Poly goat	1:30	Digestion with 0.1% Pepsin/0.5 m acetic acid (Sigma) for 2 h (37°C)	Southern Biotech, Birmingham, AL, USA
Cytokeratin	Mouse mono, Clone AE1 + AE3	1:250	Boil in Tris–EDTA (Dako) 20 min (steamer)	Cell Marque, Rocklin, CA, USA
Vimentin	Mouse mono, Clone V9	1:200	Boil in citrate buffer 20 min (steamer)	Dako, Glostrup, Denmark
Osteocalcin	Poly rabbit	1:400	No pre‐treatment	AbD Serotec, Oxford, UK (Bio‐Rad Company)

As chromogen 3, 3′diaminobenzidine‐tetrahydrochloride (Sigma‐Aldrich) substrate in Tris buffer pH 7.4 was used, slides were washed in distilled water, counterstained with haemalumn, dehydrated and subsequently mounted by the use of xylene‐soluble medium DPX (Fluka, Buchs, Switzerland). Fluorescent labelling was performed with Alexa Fluor 488 secondary antibodies (Molecular Probes, Eugene, OR, USA) for anti‐collagen I, anti‐cytokeratin, anti‐vimentin and anti‐osteocalcin (antibody sources and dilutions, see Table 1). Fluorescent counterstaining of cell nuclei was detected with 4′, 6‐diamidino‐2‐phenylindole (DAPI; Molecular Probes). For negative controls, the primary antibody was replaced by PBS solution. Photomicrographs were taken with Zeiss Axioskop Imager Z.2 microscopy (Carl Zeiss, Vienna, Austria) and analysed using zen 2012 software (Carl Zeiss).

Determination of the Ki‐67 index was performed for 3D spheroids (7, 14 and 19 day) and 2D monolayer (2 and 7 day) samples. All cells with nuclear labelling were assessed as Ki‐67 positive. Sections from the respective different cultivation time points and the four biological replicates were evaluated. The Ki‐67 index reflects the percentage of positive cells compared to a total cell number (Maglennon et al., 2008). To reach a total cell number of 700–1100 cells/Rp, randomly selected high power fields (400 × 0.065 mm^2^) from three to five slides were evaluated. The final index value of each cultivation time point was represented as mean percentage value ± SD (Table S1).

### Transmission electron microscopy

Cell pellets from 2D and 3D cultures were fixed in 3% buffered glutaraldehyde (pH 7.4) and stored at 4°C for a minimum of 12 h. Samples were flushed gently three times with 1 m phosphate buffer (according to Sörenson, pH 7.4), post‐fixed in 1% osmium tetroxide (Electron Microscopy sciences, Hatfield, PA, USA) for 2 h at RT, washed and dehydrated by immersion in an ethanol series. The infiltration with propylene oxide was followed by increasing ratios of epoxy resin : propylene oxide (1:1, 3:1). Finally, samples were put in pure epoxy resin (Serva, Mannheim, Germany) and polymerized at 60°C for 48 h. Semithin sections of 0.8 *μ*m were cut at an ultramicrotome (Reichert Ultracut S; Leica, Vienna, Austria) and stained with toluidine blue. Ultrathin sections (70 nm) were contrasted with alkaline lead citrate (Merck, Darmstadt, Germany) and methanolic uranyl acetate (Sigma‐Aldrich). Evaluation of the ultrathin sections was assessed with a Zeiss EM 900 transmission electron microscope and a digital frame‐transfer CCD camera (Tröndle TRS, Moorenweis, Germany) with siS software (ImageSP Professional; Tröndle TRS).

## Results

### Monolayer cell culture

**Figure 1:**
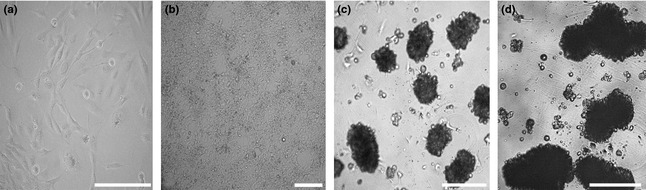
Examples of cell culture phase contrast light microscopy images of D17 canine osteosarcoma cells cultivated as 2D monolayers (a, b) or three‐dimensional (3D) spheroids (c, d). Monolayer cultivated to near confluence after 2 days. Within the monolayer lawn, epithelial‐like cells were mainly observed; occasional spindle‐shaped cells were located within less confluent areas (a). 2D monolayer after 7 days exhibited an extremely dense cell lawn (b). Spheroids developed the characteristic 3D architecture as round‐shaped cell conglomerates within the first 7 days of cultivation (c). After cultivation for 14 days, spheroids exhibited increased diameters with tightly arranged cells (d). Scale bars = 200 *μ*m.

After seeding, D17 cells were adherent within 3–5 h. In the 2D monolayer system, D17 OS cells formed 70–80% confluent cell lawns after 2–3 days after plating in 25‐cm² tissue flasks (Fig. 1a). In the first 2 days, D17 cells displayed partly epithelial‐like shaped cells and partly spindle‐shaped morphology which developed until day 7 into a very dense epithelial‐like cell lawn with areas of overgrowing cells (Fig. 1b).

## 3D spheroid cell culture

**Figure 2:**
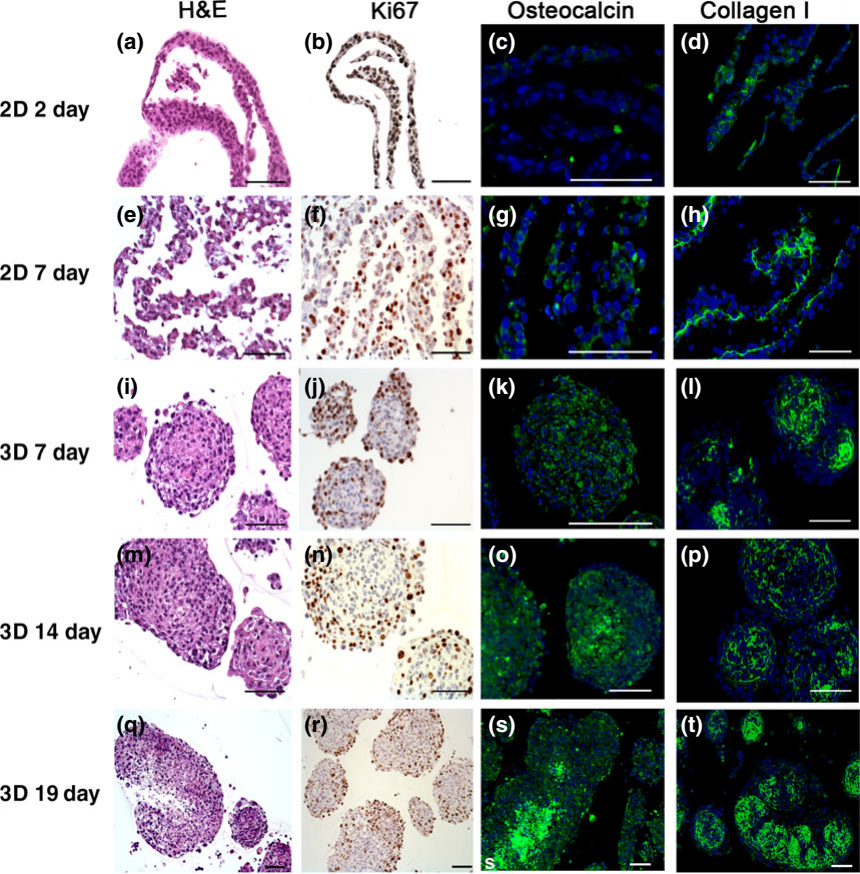
Histochemical and immunohistochemical staining on 2D monolayer (a–h) and three‐dimensional spheroid (e–t) cell culture. The illustrated samples were stained for H&E (a, e, j, m, q) as well as immunostained for Ki‐67 proliferation marker (b, f, j, n, r), osteocalcin (c, g, k, o, s) as an indicator for calcification and collagen I (d, h, l, p, t) for fibre demonstration. Scale bars = 100 *μ*m.

In the 3D system, D17 cells started to form cell clusters within 24 h after seeding. After 48 h, we observed only low amounts of non‐adherent cells in the growth medium. The majority of cells were arranged to cell islets or loose cell aggregates. The characteristic 3D structure was established after 4 day in culture displayed by compact round‐shaped spheroids with a mean size of 120 *μ*m. A continuous increase in the spheroid diameter was observed from days 7 to 19 (Figs 1c,d and 2). The mean length–diameter proportion of 7‐day spheroids was 188.2 ± 97.9 *μ*m. After 14 day, spheroids grew continuously to a mean size of 252.4 ± 106.3 *μ*m, whereas after 19 day, the mean size was enhanced to 377.7 ± 164.6 *μ*m. Overall, a heterogenic spheroid‐size pattern developed after 14 and 19 days of 3D culture.

### Histochemistry

Results of the histochemical and immunohistochemical stainings are summarized in Table 2. Images from H&E staining of 2D cultivated cells (2 day) exhibited mostly epithelial‐like cells with little amount of spindle‐shaped cells (Fig. 2). The histological pattern of the 7‐day culture originally grown as monolayer showed predominantly epithelial‐like cells featuring areas with two or more layers (Fig. 2).

**Table 2:** Evaluation of the histological staining results of D17 osteosarcoma cells grown as two‐dimensional (monolayer) and three‐dimensional (spheroid) cell culture

	Monolayer 2 day	Spheroid 7 day	Spheroid 14 day	Spheroid 19 day
Histochemical staining				
Aniline blue	−/+	+	+	+
Periodic acid–Schiff	−/+	+	+	+
Safranin O	−	−	−	−
Immunohistochemical staining				
Alkaline phosphatase	+	+	+	+
Ezrin	+	+	+	+
S100A4	+	+	+	+
Ki‐67	+	+	+	+
Collagen I	−/+	+	+	+
Cytokeratin	−	−	−	−
Vimentin	+	+	+	+
Osteocalcin	−/+	−/+	+	+

Scoring of the histochemical and immunohistochemical staining pattern: positive immunoreaction (+); no positive immunoreaction (−); (−/+) ranging from no positive to minor immunoreaction.

The H&E staining of 2D monolayer cultures and 3D spheroids after cultivation of 7, 14 and 19 day displayed characteristic tumour cell characteristic nuclei. Epithelial‐like cells in spheroids were densely arranged in the inner area and partly flattened at the spheroid periphery through all cultivation periods. Spheroids cultivated for 7 and 14 day showed vital cells in spheroid centres, while 19‐day spheroids showed clear signs of central necrosis (Fig. 2). Necrotic phenomena started in the core region of cultivated spheroids with diameters above 300 *μ*m.

**Figure 3:**
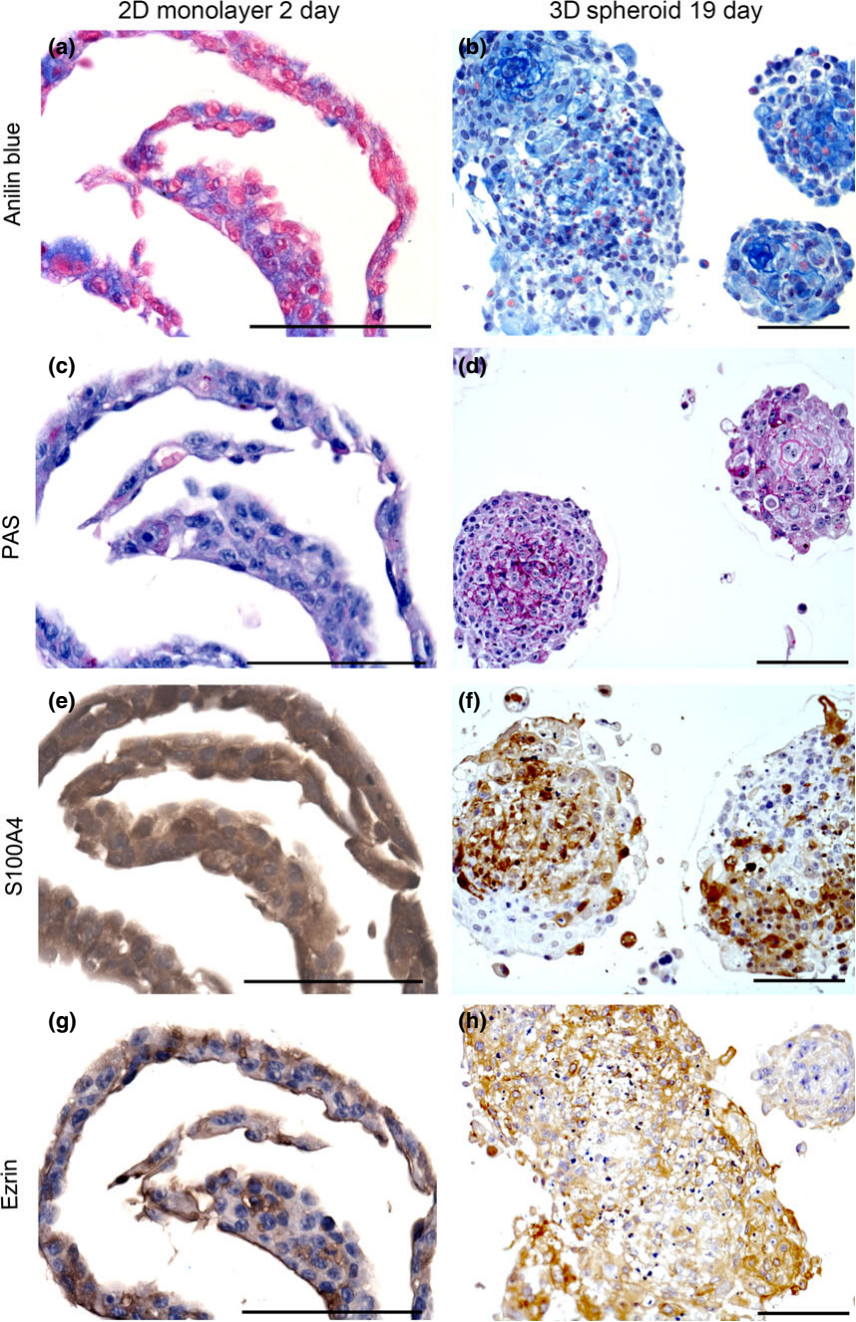
Histochemical analysis of sections of two‐dimensional (2D) monolayer cell cultures (a, c, e, g) and three‐dimensional (3D) spheroids (b, d, f, h). Samples were stained for aniline blue (a, b) and periodic acid–Schiff (PAS) (c, d). Note the delicate network of extracellular matrix positive for PAS and aniline blue, particularly in 3D spheroids. Immunohistochemical detection of S100A4 (e, f) and ezrin (g, h) in 2D and 3D cultures. Scale bars = 100 *μ*m.

Cell culture sections from 2D and 3D samples were histochemically stained for aniline blue, PAS and safranin O. For aniline blue and PAS, 2D samples displayed a weak staining pattern; only small areas of ECM and glycoprotein‐rich inter‐cellular substance were observed (Fig. 3). A more distinct staining was observed for aniline blue and PAS in particular in the inter‐cellular space in the centres of spheroids (Fig. 3). No positive signals were seen in 3D and 2D samples with safranin O staining indicating the absence of cartilage‐like matrix (not shown).

### Immunohistochemistry

We applied immunohistochemistry to further analyse components of the ECM, cell proliferation, tumour and metastasis markers. Collagen type I was occasionally observed in monolayer cell culture after 2 day in culture; in samples of canine OS cells after 7 day, monolayer culture collagen fibres were arranged as a layer at the basal side of the cell lawn (Fig. 2). In the 7‐ and 14‐day spheroids, a relevant amount of collagen I was present, forming a distinct network between the OS cells. Strongest signals for collagen I immunostaining were observed in 19‐day spheroids (Fig. 2).

Proliferative activity was conducted by anti‐Ki‐67 immunohistochemistry (Fig. 2). In 2D cell cultures, positive stained nuclei were distributed regularly within the cell layer (2 and 7 day). Mean percentage levels for Ki‐67 of 2D samples after 7 day of cultivation were 38.7 ± 4.3% compared to the slightly lower values of samples cultivated for 2 day by 36.0 ± 3.3%. In 3D cultures, the majority of Ki‐67‐positive cells were located on the periphery of spheroids. Highest mean values were found in 7‐day spheroids with 39.8 ± 7.6% decreasing from 14‐day spheroids (36.8 ± 13.1%) to 19‐day spheroids (22.6 ± 5.7%).

The two metastasis markers ezrin and S100A4 (metastasin) were present in the cytoplasm of all 2D and 3D samples (Fig. 3). Ezrin‐positive cells were irregularly distributed in monolayer cultures. In spheroids, ezrin‐positive cells were mainly located in the peripheral areas. Larger necrotic areas as seen in 19‐day spheroids showed reduced ezrin immunoreactivity (Fig. 3). Immunostaining of S100A4 showed intense signals in 2D and 3D culture sections. In monolayers, the distribution was rather regular, whereas in the spheroids, a patchy pattern of positive cells was observed including spheroid necrotic central regions (Fig. 3). Strong immunocytochemical signals were detected in all samples for vimentin as a mesenchymal cytoskeletal marker. No expression of cytokeratin was observed in any OS cell culture whether 2D or 3D (not shown). Osteocalcin and alkaline phosphatase (not shown) were identified in all samples. In 2D monolayers cultured for 2 day, only a minor positive reaction for osteocalcin was observed; this immunoreactivity increased after 7 day of cultivation (Fig. 2). Osteocalcin was found regularly distributed in 7‐day spheroids, but in 14‐ and 19‐day spheroids, osteocalcin was mainly restricted to central necrotic areas (Fig. 2).

### Transmission electron microscopy

**Figure 4:**
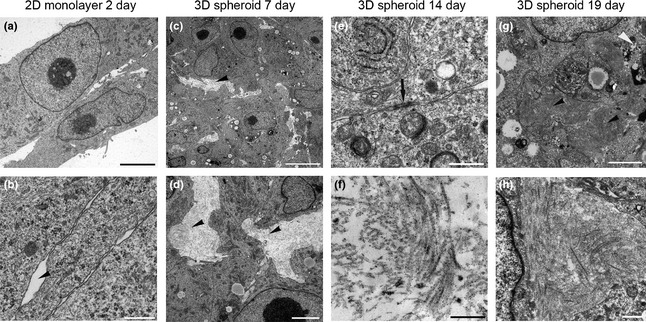
Transmission electron microscopy images of two‐dimensional (2D) monolayers and three‐dimensional (3D) spheroids generated from canine osteosarcoma cells (D17). Characteristic 2D monolayer cells cultivated for 2 days show tight cell contacts lacking signs of extracellular matrix structures between cells (a, b). Scale bars = 5 *μ*m. Inter‐cellular space marked by arrowhead (b). Note viable tumour cells with characteristic nuclei with the prominent nucleoli in spheroids cultivated for 7 day (c). Inter‐cellular space provided with collagen; arrowheads (c, d). Scale bar = 2.5 *μ*m. Undefined electron‐dense agglomerates at the cell membranes with tight cell–cell contacts within the spheroids (black arrow) in spheroids after 14 day in culture (e). Collagen I fibres were clearly identified in inter‐cellular space of spheroids cultivated for 14 day (f). Scale bar = 0.5 *μ*m. After 19 days of culture, the amount of lipid droplets as well the amount of necrotic cells (black arrowhead) was increased (g). Scale bar = 2.5 *μ*m. Densely arranged collagen I fibres predominated the extracellular space in 19‐day spheroids (h). Scale bar = 0.5 *μ*m.

TEM analysis from 2D monolayer samples showed nuclei as characteristic for tumour cells, predominated by euchromatic structure with large nucleoli. Cell membranes were intact and fairly tightly connected with adjacent cells leaving narrow inter‐cellular space (Fig. 4a,b). At the basal side of the monolayer, proteoglycans and collagen fibres were observed in samples cultivated for 7 day. The amount of lipid droplets was fairly low. Endocytosis vesicles were observed regularly at the cell membranes. Apoptotic cells were hardly seen as well as large necrotic areas were missing.

TEM analysis of 3D spheroid samples (7, 14 and 19 day) revealed well‐developed cell organelles such as endoplasmic reticulum, mitochondria and Golgi apparatus. Clathrin‐coated vesicles were frequently present at the membrane; lipid droplets were occasionally observed. Nuclei displayed the typical shape of neoplastic cells with a loose chromatin structure and a large electron‐dense nucleolus as seen in monolayer cultures. Cells were tightly arranged by interdigitating with microplicae to neighbouring cells and membranes tightly arranged, leaving almost no inter‐cellular space. Although the neighbouring cell membranes were approaching directly, no specific cellular adhesion junctions were observed. In areas where a wider inter‐cellular space was present, it was provided in different density with collagen fibres (Fig. 4c,d). The spheroids cultivated for 7 day rarely exhibited apoptotic cells.

The ultrastructure of the spheroids from 14 day was quite similar to the 7‐day cultures. Generally, the inter‐cellular spaces were wider with a prominent amount of collagen type I fibres (Fig. 4f). Furthermore, an unidentified electron‐dense ECM component was detected, and cell membranes were closely arranged in areas of these adhesion‐related structures (Fig. 4e). The amount of lipid droplets was obvious compared to the 7‐day spheroids; also, the area of necrosis was evident in the core region of larger sized spheroids. At the cell surfaces, pinocytotic vesicles were frequently present.

After 19 day of cultivation, spheroids showed considerable amounts of lipid droplets and necrotic cells compared to spheroids of 7 and 14 day (Fig. 4g). Distinct necrotic areas were observed in the centre of larger spheroids. Considerable amounts of collagen I fibres were present (Fig. 4h). Other cell characteristics such as abundance of cell organelles and cell contacts remained unchanged.

## Discussion

Over the last decades, much effort has been made to establish novel 3D cell culture models with the aim to obtain more relevant results from *in vitro* studies in particular for drug discovery and biomarker research. There are several advantages for applying cell culture in cancer research such as the reduction of animal studies which are expensive, labour‐intensive and worthy for discussion due to ethical aspects. In contrast to classical monolayer cell culture, 3D cell models are desirable as they are highly comparable to the *in vivo* tumour tissue. Nowadays, one important method of choice is 3D spheroids which highly mimic the *in vivo* morphology, and functional and transport features of the corresponding tumour tissue (Levinger et al., 2014).

Several attempts for 3D cultures have been made in OS research. Critical mechanism of OS chemoresistance has been demonstrated in eleven cell lines cultivated as monolayer and spheroid systems (Arai et al., 2013). Their findings revealed a noticeable decreased drug response in 3D cells compared to 2D grown cells, which represents well the *in vivo* situation for OS treatment. In further studies, the ability of murine OS 3D alginate spheroid cultures to generate lung metastases has been reported (Akeda et al., 2009). Human OS cells have been successfully cultured using a porous silk sponge (Tan et al., 2011). Comparing different angiogenic factors, these authors could demonstrate a significant difference between 2D monolayer and 3D silk scaffold systems. We followed these attempts by focusing on 3D spheroids in OS cell culture. It is important to apply a well‐characterized cell culture system which assures the optimum usability.

Canine D17 OS cells were suitable for spontaneous spheroid formation in non‐adherent round‐bottom 96‐well plate format obtaining reproducible results. Our data depicted a significant increase in spheroid size over the cultivation period (7, 14 and 19 day). Murphy et al. (2014) described that spheroid size differs significantly according to the employed cell number. However, smallest spheroids showed highest metabolic activity and significantly lower amounts of apoptotic signs compared to large size spheroids. It has to be considered that these were short‐term cultures (48 h) compared to our long‐term cultures for up to 19 day. These results support our findings as we determined lower amounts of necrotic cells in spheroids from 7 day compared to 19 day. In 19‐day spheroid cultures, we commonly observed necrotic areas arising in the centre of the spheroids. The development of necrotic cores started in spheroids with diameters of more than 300 *μ*m surrounded by a rim of viable and proliferative cells. We generally detected a proliferation gradient in spheroids from the centres to the periphery. This phenomenon has been analysed by Grimes et al. (2014) who evaluated oxygen consumption rate, the diffusion limit as well as the hypoxic region in tumour spheroids. Overall, they found a relatively constant diffusion limit of 232 ± 22 *μ*m spheroid diameter. Oxygen diffusion was detected in the peripheral region in the location of the proliferating rim, whereas no oxygen supply was measured in the inner zone in the necrotic core region. This is in accordance with the outcome from Kunz‐Schughart et al. (2004) who reported cell‐cycle‐arrested cells at larger distances from the surface and a necrotic core in most spheroids larger than 400–500 *μ*m. It is likely that due to the increasing size of spheroids, unfavourable conditions, including hypoxia within the core occur and lead to the regularly observed cell necrosis. Lin and Chang (Lin and Chang, 2008) described a characteristic three‐layer structure of large size spheroids (>500 *μ*m) including a necrotic core, surrounded by a quiescent viable cell zone and an outer proliferating zone. The phenomenon of centre necrosis also occurs in tumours and metastasis when they reach sizes above 500 *μ*m (Grimes et al., 2014). Metastases have to resist the conditions of hypoxia, inflammation, changes in pH levels and often nutrient deprivation. Moreover, hypoxia itself increases the likelihood of metastasis development and drug resistance (Grimes et al., 2014). As a consequence, the tumour cells express pro‐angiogenic factors to enable vascularization and provision with nutrients and oxygen (Ghesquière et al., 2014). Based on these reports, we suppose that our spheroid model is suitable to study the hypoxic part of a non‐vascularized OS tumour. In parallel to the development of necrotic areas during culture time, the amount of lipid droplets increased. The accumulation of lipid droplets is interpreted as sign of degeneration, however, could also be due to inadequate medium composition.

We compared the two cell culture systems regarding the synthesis of ECM such as glycoproteins (determined by PAS and aniline blue histochemistry) and collagen I (determined by immunohistochemistry). Both culture systems stimulated collagen I production in canine D17 OS cells; however, the distribution pattern of collagen I in 2D cell culture and spheroids was completely different. The arrangement in the 3D culture mimicked the *in vivo* fibre network closely (Broadhead et al., 2011). Tumour growth is triggered by cell–ECM interaction. Collagen I as ECM component has a predominant role in tumour invasion via activating remodelling factors such as MMP2 (Elenjord et al., 2009; De Wever et al., 2014). Another report support our findings with enhanced expression of *COL1A1* mRNA levels in human OS cells (SaOS2) particularly in long‐term 3D cell culture (Prideaux et al., 2014). Safranin O, as cartilage protein marker, revealed no positive staining results indicating the absence of cartilage‐like differentiation of the OS cells in both culture systems.

Both 2D and 3D cell cultures produced the osteogenic marker osteocalcin. A colocalization of osteocalcin with necrotic areas of spheroids was observed. Nishida et al. (2000) observed enhanced mRNA levels of alkaline phosphatase, osteopontin and osteocalcin in humans in SaOS‐2 and MC3T3‐E1 cells. Canine appendicular OS and high levels of serum total alkaline phosphatase as well as bone‐related alkaline phosphatase are directly related with poor survival time and less disease‐free intervals (Ehrhart et al., 1998; Garzotto et al., 2000; Kirpensteijn et al., 2002). Furthermore, a significant correlation between OS tumour size and levels of bone‐specific alkaline phosphatase has been reported (Sternberg et al., 2013). In contrast to our results, Dey et al. (2014) observed a lack of alkaline phosphatase expression under monolayer culture conditions in human U2OS and SaOS‐2 OS cells. However, when cultivated on chitosan–nHA polymer matrix, alkaline phosphatase was detectable. This might be due to species differences or different culture conditions (2D, 3D) including cell density.

We found two metastasis markers ezrin and S100A4 (metastasin) expressed in both 2D and 3D cell culture systems. Ezrin has a crucial role in OS as cell signalling, cell interaction and metastasis development marker (Zhang et al., 2014), and S100A4 (metastasin) is one of the key markers in promoting metastasis development in OS (Cao et al., 2014). It has been reported for canine OS that increased ezrin levels showed significantly shorter median disease‐fee intervals (Ren and Khanna, 2014). The authors concluded that the expression of an enhanced ezrin level may directly contribute to metastasis formation. In the present study, no remarkable differences in ezrin or S100A4 expression were observed neither between culture time points nor cell culture systems.

Our results from light and TEM revealed remarkable close contacts with frequent interdigitations between neighbouring cells within the spheroids during all time points (7, 14 and 19 day). However, we could not identify specific cellular junctions on the structural level. Ezrin as membrane–cytoskeletal linker protein interacts with β4‐integrin and therefore promotes metastasis development (Wan et al., 2009). It has been demonstrated that integrin and ezrin form connections in human OS cell lines (Wan et al., 2009). These adherence junctions have not been identified for canine OS cells so far; however, a contribution of integrins to cell adherence in OS spheroids is likely. The expression of intermediate filaments of the cultured OS cells was not influenced by the different cell culture conditions and thereby preserved the typical mesenchymal marker profile (vimentin‐positive, cytokeratin‐negative) identified by means of immunohistochemistry.

In conclusion, the present 3D scaffold‐free cell culture model reflects the favourable properties to mirror the native *in vivo* OS architecture with autologous matrix production without the need of an artificial ECM. The further advantage of our 3D spheroid system is using a commercially available canine OS cell line that spontaneously forms 3D spheroids. Furthermore, our technique is highly reproducible, cost–effective and well adaptable, and assumed to be suitable for drug and hypoxia testing assays, tumour biology research and biomarker identification such as proteomics or genomics experiments.

## Source of Funding

This investigation was financially supported by the Austrian Science Fund (FWF), Grant Number P 23336‐B11.

## Conflict of Interest

The authors declare no conflict of interests.

## Supporting information


**Table S1.** Distribution of Ki67 positive cells.
