# Supplementary material for: Morphological and Immunohistochemical Characterization of Canine Osteosarcoma Spheroid Cell Cultures
Source: Anat Histol Embryol. 2015 Aug 19;45(3):219–30. doi: 10.1111/ahe.12190 (PMC4949528; doi:10.1111/ahe.12190)
Supplement: Supplementary file 1 — Table S1. Distribution of Ki67 positive cells. [file AHE-45-219-s001.doc]

**Supplementary Table 1:** Distribution of Ki67 positive cells

| **3D Spheroids** | | | |
| --- | --- | --- | --- |
| *Sample No.* | *Total cell No.* | *Mean (%) Ki67 positive ± SD* | *Rp1-4: Mean (%)  Ki67 positive ± SD* |
| 7d/Rp 1 | 738 | 36.0 ± 0.6 | 39.8 ± 7.6 |
| 7d/Rp 2 | 920 | 37.4 ± 10.0 |
| 7d/Rp 3 | 673 | 47.1 ± 6.0 |
| 7d/Rp4 | 806 | 37.6 ± 6.0 |
| 14d/Rp 1 | 737 | 28.6 ± 5.4 | 36.8 ± 13.1 |
| 14d/Rp 2 | 881 | 48.4 ± 17.4 |
| 14d/Rp 3 | 953 | 32.1 ± 6.2 |
| 14d/Rp4 | 1110 | 33.5 ± 4.0 |
| 19d/Rp 1 | 910 | 21.1 ± 4.9 | 22.6 ± 5.7 |
| 19d/Rp 2 | 781 | 19.0 ± 1.6 |
| 19d/Rp 3 | 930 | 26.8 ± 7.6 |
| 19d/Rp4 | 1103 | 23.0 ± 5.8 |
|  |  |  |  |
| **2D Monolayer** | | | |
| *Sample No.* | *Total cell No.* | *Mean (%) Ki67 positive ± SD* | *Rp1-4: Mean (%)  Ki67 positive ± SD* |
| 2d/Rp1 | 831 | 35.9 ± 5.5 | 36.0 ± 3.3 |
| 2d/Rp2 | 652 | 36.7 ± 2.9 |
| 2d/Rp3 | 699 | 34.4 ± 5.5 |
| 2d/Rp4 | 940 | 37.0 ± 0.8 |
| 7d/Rp1 | 835 | 35.1 ± 0.2 | 38.7 ± 4.3 |
| 7d/Rp2 | 874 | 39.0 ± 6.1 |
| 7d/Rp3 | 914 | 38.0 ± 4.2 |
| 7d/Rp4 | 1069 | 42.7 ± 4.2 |

Sample No.=Sample number; Total cell No.=Sum of the counted cells per biological replicate (Rp); SD=standard deviation
